# Correlative Localization Analysis Between mRNA and Enhanced Green Fluorescence Protein-Fused Protein by a Single-Molecule Fluorescence *in situ* Hybridization Using an *egfp* Probe in *Aspergillus oryzae*

**DOI:** 10.3389/ffunb.2021.721398

**Published:** 2021-10-13

**Authors:** Yuki Morita, Yoshinori Katakura, Kaoru Takegawa, Yujiro Higuchi

**Affiliations:** Department of Bioscience and Biotechnology, Faculty of Agriculture, Kyushu University, Fukuoka, Japan

**Keywords:** *Aspergillus oryzae*, enhanced GFP, filamentous fungi, mRNA localization, single-molecule FISH

## Abstract

Although subcellular localization analysis of proteins fused with enhanced green fluorescence protein (EGFP) has been widely conducted in filamentous fungi, little is known about the localization of messenger RNAs (mRNAs) encoding the EGFP-fused proteins. In this study, we performed single-molecule fluorescence *in situ* hybridization (smFISH) using an *egfp* probe to simultaneously visualize EGFP-fused proteins and their mRNAs in the hyphal cells of the filamentous fungus *Aspergillus oryzae*. We investigated the subcellular localization of mRNAs encoding cytoplasmic EGFP, an actin marker protein Lifeact tagged with EGFP, and several EGFP-fused proteins AoSec22, AoSnc1, AoVam3, and AoUapC that localize to the endoplasmic reticulum (ER), the apical vesicle cluster Spitzenkörper, vacuolar membrane, and plasma membrane, respectively. Visualization of these mRNAs by smFISH demonstrated that each mRNA exhibited distinct localization patterns likely depending on the mRNA sequence. In particular, we revealed that mRNAs encoding Lifeact-EGFP, EGFP-AoSec22, EGFP-AoVam3, and AoUapC-EGFP, but not cytoplasmic EGFP and EGFP-AoSnc1, were preferentially localized at the apical cell, suggesting certain mechanisms to regulate the existence of these transcripts among hyphal regions. Our findings provide the distinct localization information of each mRNA in the hyphal cells of *A. oryzae*.

## Introduction

The filamentous fungus *Aspergillus oryzae* has been utilized in the fermentation and brewing industries because it can safely produce copious amounts of useful enzymes (Kitamoto, 2015; Ichishima, 2016; Gomi, 2019; Kitagaki, 2021). Due to the great ability of enzymatic protein secretion, the molecular machinery of membrane traffic, including the secretory pathway, has been well-investigated in *A. oryzae* (Shoji et al., 2014; Higuchi, 2021a,b). For example, 21 soluble *N*-ethylmaleimide-sensitive factor attachment protein receptors (SNAREs) that have a crucial role in vesicular trafficking have been identified, and enhanced green fluorescence protein (EGFP)-fused SNARE proteins have been analyzed for understanding this subcellular localization (Kuratsu et al., 2007). According to the analysis, the endoplasmic reticulum (ER), visualized by a SNARE protein AoSec22 tagged with EGFP, was found to exhibit meshwork-like structures in the hyphal cells of *A. oryzae* (Kuratsu et al., 2007; Morita et al., 2020). In addition, a vesicle SNARE AoSnc1 is predominantly localized to the apical vesicle cluster Spitzenkörper (Togo et al., 2017). On the other hand, endocytosis has been investigated using the EGFP-fused plasma membrane purine transporter AoUapC (Higuchi et al., 2006, 2009b). Moreover, visualization of the vacuolar membrane with the EGFP-fused SNARE AoVam3 demonstrated that vacuoles are highly dynamic with different shapes in each region of hyphae (Shoji et al., 2006). Although these EGFP-fused protein analyses revealed molecular mechanisms of membrane traffic in *A. oryzae*, such information so far is mainly based on the protein level, and thus spatiotemporal molecular machinery of when and where proteins are translated is largely unknown.

To elucidate the subcellular locations where proteins are synthesized, mRNA localization is crucial to be investigated. The underlying mechanisms of mRNA localization have been clarified in varieties of organisms (Buxbaum et al., 2015; Mofatteh and Bullock, 2017). To analyze mRNA localization, single-molecule fluorescence *in situ* hybridization (smFISH) has been widely applied to many organisms (Raj et al., 2008; Eliscovich et al., 2017; Tutucci et al., 2018). Although cell biological expression analysis by the GFP reporter system was conducted in the filamentous fungus *Aspergillus niger* (Vinck et al., 2011; Tegelaar et al., 2020), only a few types of research by smFISH to directly visualize mRNA molecules have been reported in filamentous fungi. For instance, in *Ashbya gossypii*, the G1 cyclin transcript was investigated to demonstrate cell-cycle mechanisms (Lee et al., 2013).

Previously, we established the smFISH experiment to investigate the subcellular localization of mRNAs encoding α-amylase and actin in the hyphal cells of *A. oryzae* (Higuchi and Takegawa, 2020). For the smFISH, we employed probes specific to sequences of α-amylase-encoding *amyB* and actin-encoding *actA*. By the addition of maltose that is an inducing molecule of *amyB* expression to the culturing medium, *de novo* synthesis of *amyB* mRNAs was observed throughout the hyphal cell, suggesting that α-amylase proteins might be produced in each hyphal cell. Moreover, we revealed that *amyB* mRNAs are excluded from the hyphal tip region where α-amylase proteins are thought to be predominantly transported for secretion (Hayakawa et al., 2011). In contrast, *actA* mRNAs are localized mainly at the hyphal tip region where actin proteins are also known to be localized (Berepiki et al., 2010, 2011; Hayakawa et al., 2011). These results suggest that there are discrete subcellular localization patterns of mRNAs depending on their sequences. However, simultaneous localization analysis of mRNAs and proteins for *amyB* and *actA* remained to be elucidated.

In this study, we further applied smFISH using an *egfp* probe to investigate the subcellular localization of both EGFP-fused proteins and their mRNAs in the hyphae of *A. oryzae*. This was possible because the localization of EGFP-fused proteins was conserved even after smFISH procedures. By analyzing mRNA localization of several EGFP-fused proteins AoSec22, AoSnc1, AoVam3, and AoUapC that localize to ER, Spitzenkörper, vacuolar membrane, and plasma membrane, respectively, we revealed possible underlying mechanisms for the localization of each mRNA and protein in hyphal cells of *A. oryzae*.

## Materials and Methods

### Generation of Plasmids and Strains

*Aspergillus oryzae* wild-type strain RIB40 was used as a DNA cloning donor and negative control without EGFP. For expressing EGFP or EGFP-fused protein, *amyB* promoter (P*amyB*) was used, and an *A. oryzae* strain NSlDS1 was used as a host. A strain expressing cytoplasmic EGFP was constructed by *A. oryzae* transformation of pgPamyB-SmaI-egfp-TamyB. To construct a Lifeact-EGFP-expressing plasmid, pgPamyB-SmaI-egfp-TamyB was used as an expression vector. The DNA sequence of *Lifeact* was amplified by PCR using PrimeSTAR GXL DNA polymerase (Takara) and the primer set: YHK251, 5′- ATCAAGAAGTTCGAGTCCATCTCCAAGGTGGTGATGGTGAGCAAGGGCGAGGAG-3′; YHK252, 5′-CTCGAACTTCTTGATGAGATCGGCGACACCCATCTGTGGGGTTTATTGTTCAGAGAAGGG-3′, and then ligated with the PCR-amplified vector by In-Fusion reaction (Takara), resulting in pgLifeactegnD. For preparing an AoUapC-EGFP-expressing plasmid pgPaUAPCegnD, the abovementioned same cloning strategy with the expression vector was conducted using the primer set: AT143, 5′-TAAACCCCACAGCCCATGGATGGACCAGATCAGATTGGTCCTG-3′; AT144, 5′-CTTGCTCACCATCCCTACAATAGCCGGACCAGCCTCGG-3′. To generate strains expressing EGFP-AoSec22, EGFP-AoSnc1, or EGFP-AoVam3, plasmids pgPaGAoSec22, pgPaGAoSnc1, or pgPaGAoVam3 was introduced into the strain NSlDS1 (Togo et al., 2017; Morita et al., 2020). All strains used in this study are summarized in Table 1.

**Table 1:** Strains used in this study.

**Strain**	**Genotype**	**References**
RIB40	Wild-type	
NSlDS1	*niaD^−^ sC^−^ AosC adeA^−^ adeA* Δ*argB* Δ*ligD*::*argB*	Togo et al., 2017
PaG	*niaD^−^* (P*amyB*-*egfp niaD*) *sC^−^ AosC adeA^−^ adeA* Δ*argB* Δ*ligD*::*argB*	This study
PaLaG	*niaD^−^* (P*amyB*-*Lifeact*-*egfp niaD*) *sC^−^ AosC adeA^−^ adeA* Δ*argB* Δ*ligD*::*argB*	This study
GSec22	*niaD^−^* (P*amyB*-*egfp*-*Aosec22 niaD*) *sC^−^ AosC adeA^−^ adeA* Δ*argB* Δ*ligD*::*argB*	Togo et al., 2017
GSnc1	*niaD^−^* (P*amyB*-*egfp*-*Aosnc1 niaD*) *sC^−^ AosC adeA^−^ adeA* Δ*argB* Δ*ligD*::*argB*	Togo et al., 2017
PaUapCG	*niaD^−^* (P*amyB*-*AouapC*-*egfp niaD*) *sC^−^ AosC adeA^−^ adeA* Δ*argB* Δ*ligD*::*argB*	This study
PaGVam3	*niaD^−^* (P*amyB*-*egfp*-*Aovam3 niaD*) *sC^−^ AosC adeA^−^ adeA* Δ*argB* Δ*ligD*::*argB*	This study

### Single-Molecule Fluorescence *in situ* Hybridization

For culturing hyphal cells of *A. oryzae*, a minimal medium Czapek-Dox (CD; 0.3% NaNO3, 0.2% KCl, 0.1% KH_2_PO_4_, 0.05% MgSO_4_·7H_2_O, 0.002% FeSO_4_·7H_2_O, and 2% glucose, pH 5.5) was used. The CD liquid medium was used for microscopy, sterilized by filtering through 0.2-μm filters. Approximately 10^4^ conidia of each strain were inoculated into 100 μl of CD liquid medium, cultured in a poly-lysine-coated glass bottom dish (Matsunami), and incubated at 30°C for 20 h. smFISH was conducted with modifications to improve the sensitivity of the previous method (Higuchi and Takegawa, 2020). The following operations were performed while protected from light. Cultured cells were fixed by adding 37% formaldehyde to the culturing medium at a final concentration of 7.4% and incubated at room temperature (RT) for 1 h. These fixed cells were washed twice by 150 μl of PBS (0.8% NaCl, 0.2% KCl, 0.12% Na_2_HPO_4_, 0.2% KH_2_PO_4_, pH 6.0) with 100 mM glycine, and incubated at RT for 5 min in the second wash. To partially digest the cell wall, cells were incubated with 100 μl solution of 5 μg/μl zymolyase (Nacalai Tesque) in fixation buffer (1.2 M sorbitol, 0.1 M K_2_HPO_4_, pH 7.5) at 30°C for 1.5 h. After washing twice with 150 μl of fixation buffer, cells were incubated with 100 μl solution of 0.1% Triton X-100 (Nacalai Tesque) in fixation buffer for 10 min at RT for permeabilization. After three times wash with fixation buffer, cells were suspended in 100 μl of hybridization buffer (LGC Biosearch Technologies) containing 10% deionized formamide with or without the addition of 1 μl of 12.5 μM of the probe (final concentration of 125 nM) overnight. After replacement of the hybridization buffer with 100 μL of buffer A (LGC Biosearch Technologies) containing 10% deionized formamide, cells were incubated with 100 μL of buffer A at 30°C for 30 min. To stain the nuclei, the cells were incubated with 100 μl of buffer A containing 0.05 ng/μl DAPI (Molecular Probes) using a stock solution of 1 mg/ml in DMSO at 30°C for 30 min. Buffer A was then replaced with 100 μl of buffer B (LGC Biosearch Technologies) and incubated at RT for 3 min. The cells were then mounted in 50 μl of Vectashield Antifade Mounting Medium (Vector Laboratories) to minimize fluorescence bleaching and subsequently observed under the fluorescence microscope. The smFISH probe (LGC Biosearch Technologies) was used according to the instructions of the manufacturer. The smFISH probe for *egfp* consisted of mixtures of 18–22 nt from 37 regions in 717 bp, each region was linked to CAL Fluor Red 610 (excitation/emission, 590/610 nm). The information of the *egfp* probe sequence is summarized in Supplementary Table 1.

### Fluorescence Microscopy

An ECLIPSE Ti2-A inverted microscope (Nikon) was employed, equipped with a CFI Plan Apo Lambda 100 × objective lens (1.45 numerical aperture), a DS-Qi2 digital camera, an LED-DA/FI/TX-A triple band filter (Semrock: Exciter, FF01-378/474/575; Emission, FF01-432/523/702; Dichroic mirror, FF409/493/596-Di02), an LED light source X-LED1 and differential interference contrast (DIC) to observe the EGFP, CAL Fluor Red 610, and DAPI fluorescent signals and hyphal morphology of *A. oryzae* cells. Images were taken, merged, and processed for adjusting to the same fluorescence scaling by using the NIS Elements AR software (Nikon).

### Quantitative Analysis of Microscope Images

For determining the relative intensity, each mRNA-derived fluorescence intensity was measured at 50 μm hyphal areas of apical (from the tip), middle (septum on the center), and basal (from the germinated conidium but excluding it) regions and corrected for the background intensity taken from the extracellular space adjacent to the cell measured. Statistical analysis was performed by Tukey–Kramer test with the quantitative data of each mRNA fluorescence image (*n* = 10 cells from two independent experiments).

## Results

### Localization of mRNA Encoding Cytoplasmic EGFP

**Figure 1:**
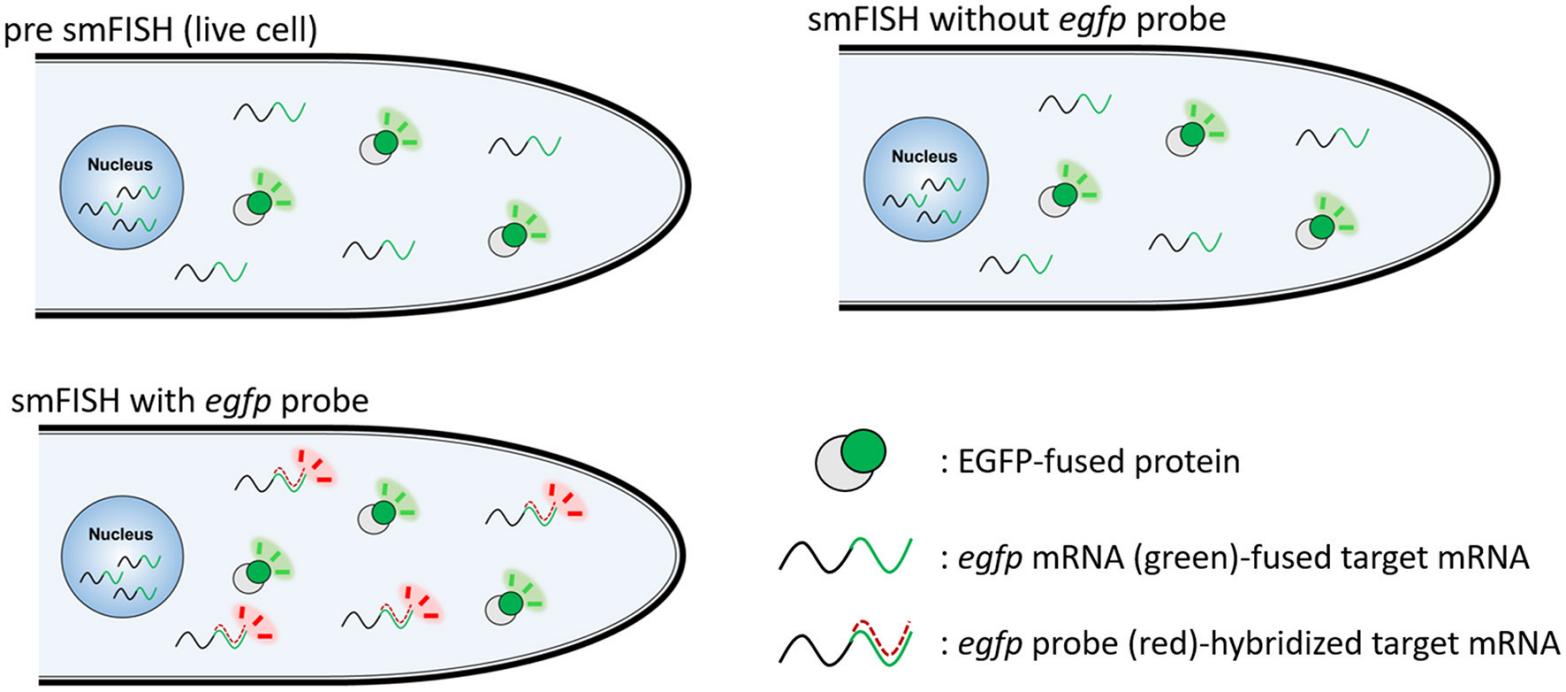
Schematic diagram to visualize EGFP-fused protein and its transcript by smFISH. EGFP-fused protein is visualized in live cells. smFISH without *egfp* probe results in the fluorescence of EGFP-fused protein. smFISH with *egfp* probe results in the fluorescence of both EGFP-fused protein and *egfp* probe-hybridized mRNA.

For visualizing *egfp*-fused mRNAs, we employed an smFISH probe based on the *egfp* sequence (Supplementary Table 1). A schematic diagram to analyze the localization of EGFP-fused protein and its transcript by smFISH using the *egfp* probe is shown in Figure 1.

**Figure 2:**
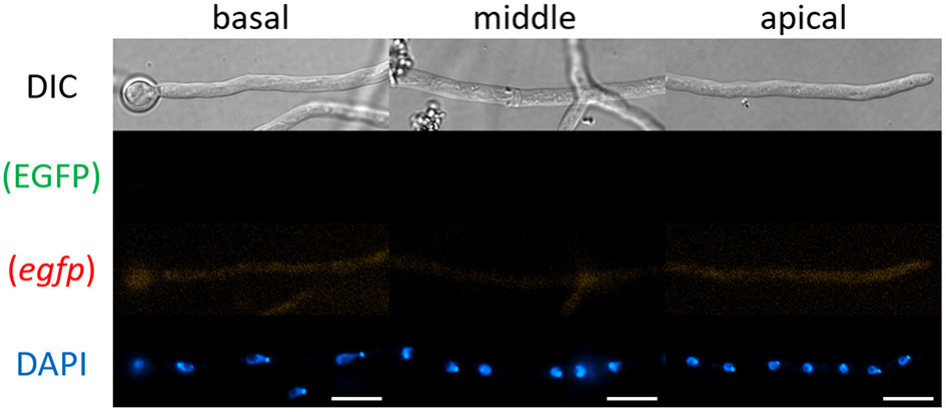
smFISH using the *egfp* probe onto cells of *A. oryzae* RIB40 wild-type strain. No EGFP and red fluorescence appeared in any hyphal regions. Nuclei were stained with DAPI. Bars, 10 μm.

First, we tested whether mRNAs encoding cytoplasmic EGFP were visualized by smFISH using the *egfp* probe. As a negative control, we performed the smFISH procedures onto cells of the *A. oryzae* wild-type strain RIB40 that does not express EGFP (Supplementary Figure 1). We found that almost no green and red fluorescence appeared in any hyphal regions (Figure 2), indicating that our smFISH microscopy settings hardy detected autofluorescence.

**Figure 3:**
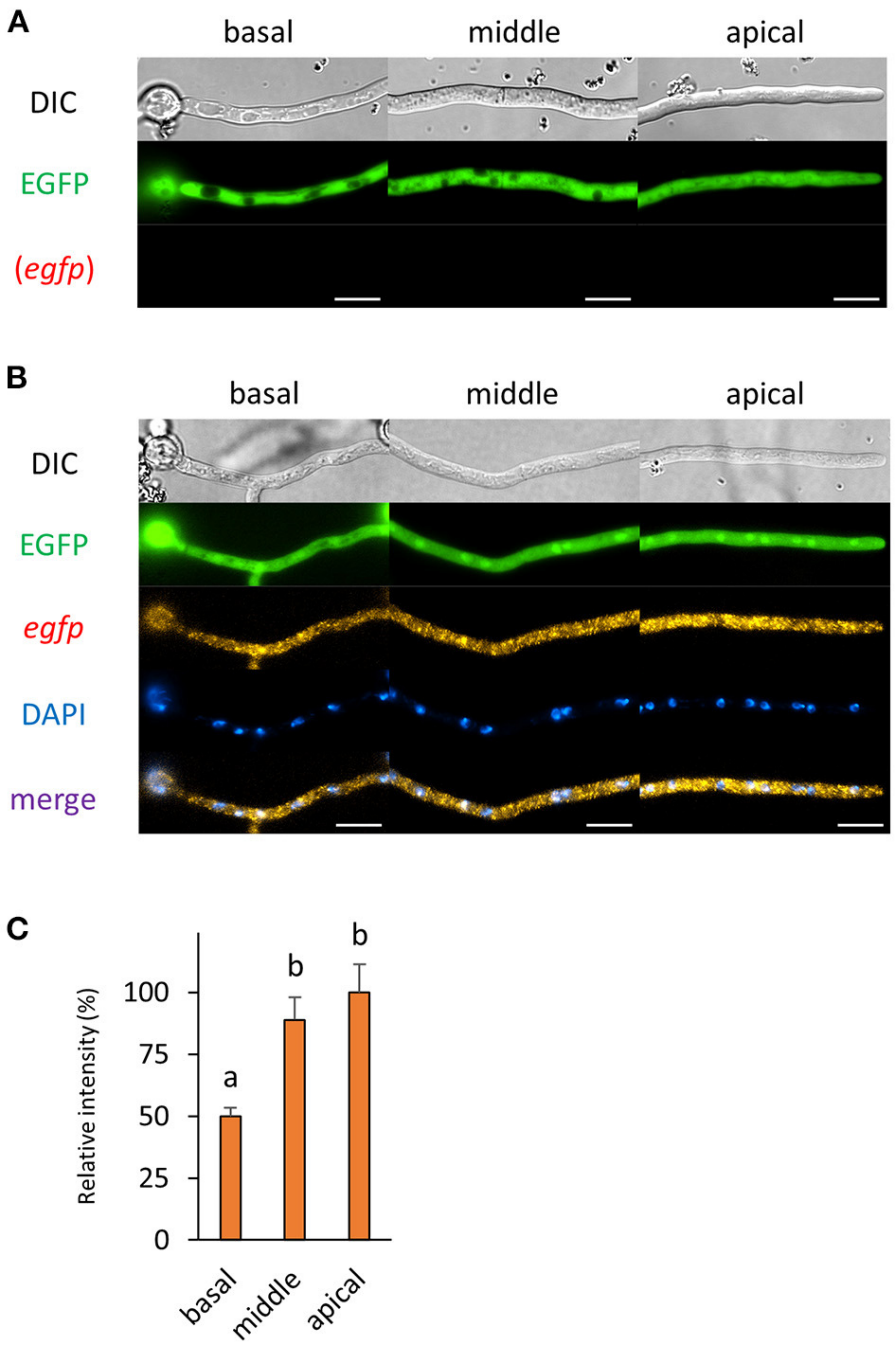
The fluorescence derived from EGFP protein and *egfp* mRNA was observed in hyphal cells of the cytoplasmic EGFP-expressing strain. **(A)** Cytoplasmic EGFP was observed in live cells. **(B)** smFISH with *egfp* probe resulted in the observation of the fluorescence of cytoplasmic EGFP and *egfp* mRNAs. Nuclei were stained with DAPI. Bars, 10 μm. **(C)** Relative fluorescence intensity of *egfp* mRNA was determined at apical, middle, and basal cells. Bars show mean with standard error. Significant difference at *P* < 0.05 between a and b (Tukey–Kramer test, *n* = 10 cells).

Then, we observed cells of the cytoplasmic EGFP-expressing strain by smFISH with the *egfp* probe. In this study, we employed the *amyB* promoter to express EGFP or its fusion protein, which enabled us to compare each mRNA localization with similar expression levels. The cytoplasmic EGFP fluorescence was consistently observed before and after smFISH procedures and in the presence or absence of the *egfp* probe (Figures 3A,B and Supplementary Figure 2A). The fluorescence of *egfp* mRNAs was seen through the cytoplasm of hyphal cells, while no red autofluorescence was seen before and after smFISH without the *egfp* probe (Supplementary Figure 2A), suggesting that the observed red fluorescence was indeed derived from *egfp* mRNA molecules with the decreased presence at the basal cell (Figure 3C).

### Localization of Lifeact-EGFP and Its Transcript

**Figure 4:**
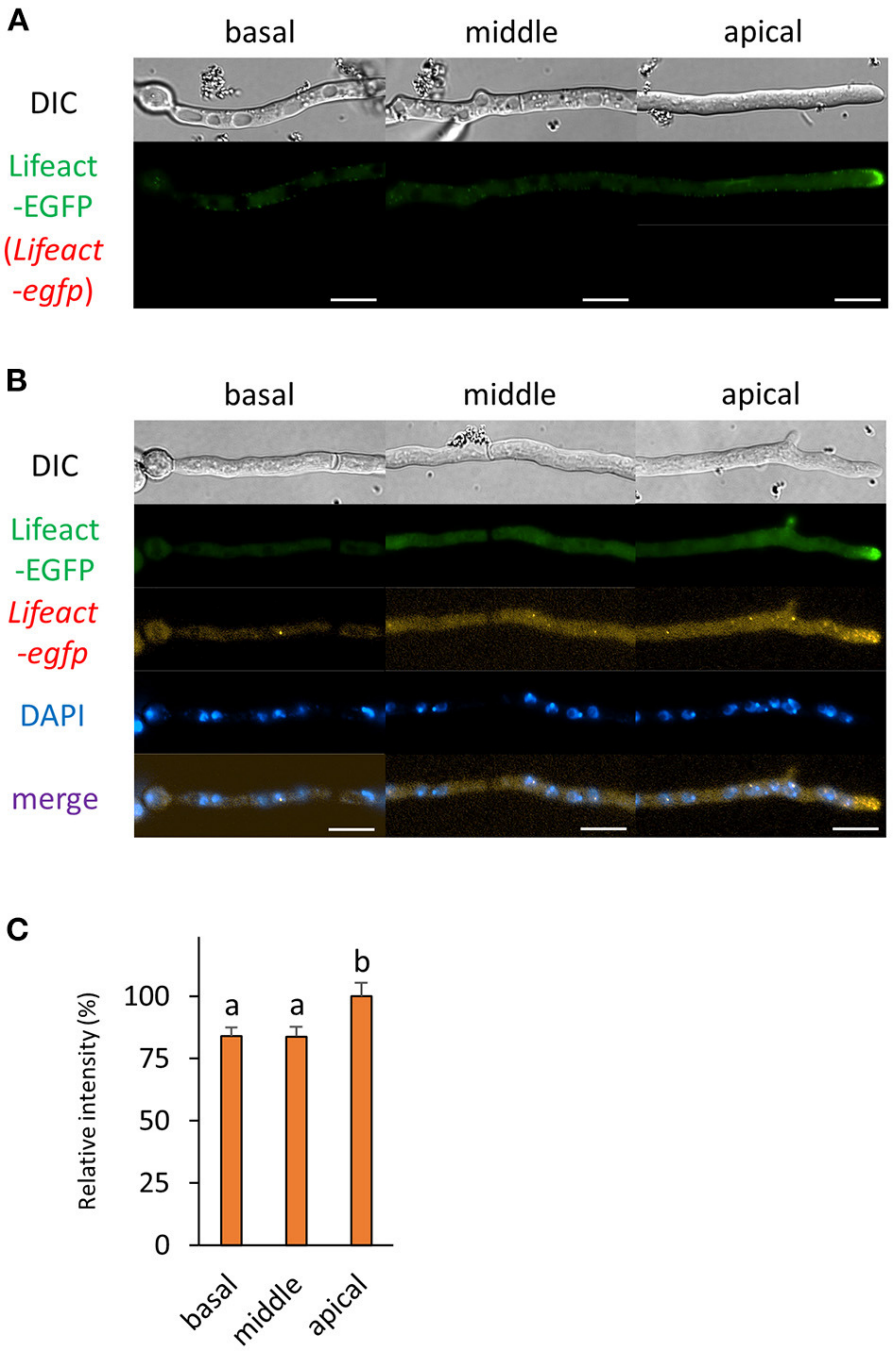
The fluorescence derived from Lifeact-EGFP protein and *Lifeact-egfp* mRNA was observed in hyphal cells of the Lifeact-EGFP-expressing strain. **(A)** Lifeact-EGFP was observed in live cells. **(B)** smFISH with *egfp* probe resulted in the observation of the fluorescence of Lifeact-EGFP and *Lifeact-egfp* mRNAs. Nuclei were stained with DAPI. Bars, 10 μm. **(C)** Relative fluorescence intensity of *Lifeact-egfp* mRNA was determined at apical, middle, and basal cells. Bars show mean with standard error. Significant difference at *P* < 0.05 between a and b (Tukey–Kramer test, *n* = 10 cells).

Previously, we analyzed the mRNA localization of *actA* that encodes actin; however, we did not investigate the localization relationship between its mRNA and protein (Higuchi and Takegawa, 2020). In this study, we employed Lifeact, a well-studied actin marker protein in a wide range of organisms, including *A. oryzae* and other filamentous fungi (Berepiki et al., 2010, 2011; Hayakawa et al., 2011). As reported previously, Lifeact-EGFP was localized especially at the tip in both living and fixed hyphal cells of *A. oryzae* (Figures 4A,B and Supplementary Figure 2B). Besides the localization in the cytoplasm, *Lifeact-egfp* mRNAs were also localized to the tip, which is consistent with the localization pattern of *actA* mRNAs (Figure 4B; Higuchi and Takegawa, 2020). Since in general there is a certain distance between the hyphal tip and nuclei, *Lifeact-egfp* mRNAs might be transported from nuclei to the tip region. Although cytoplasmic mRNA fluorescence was almost evenly distributed throughout the cell, significantly more *Lifeact-egfp* mRNAs were observed at the apical cell (Figures 4B,C).

### Localization of mRNAs Encoding EGFP-Fused SNARE Proteins that Localize to ER and Spitzenkörper

**Figure 5:**
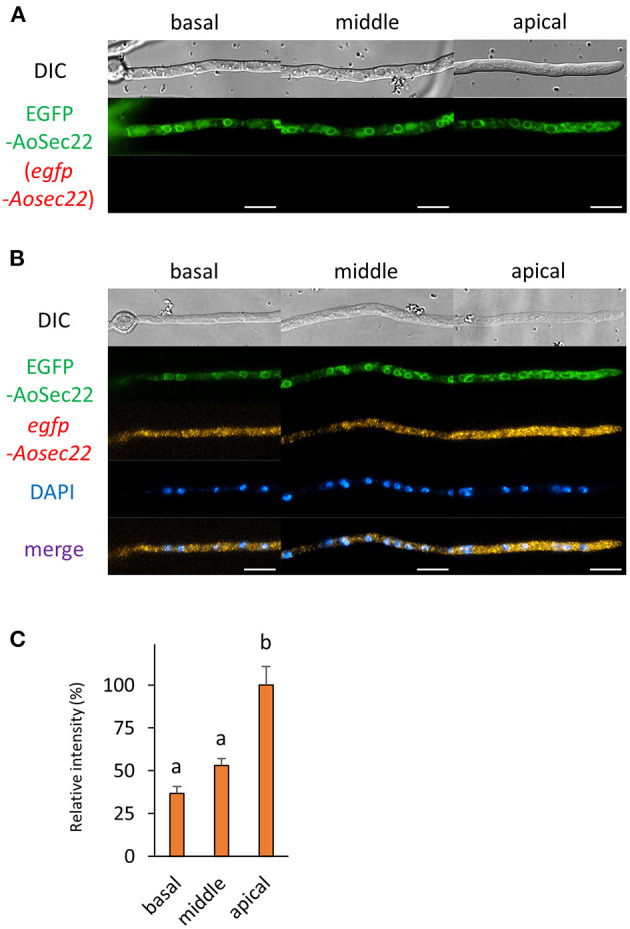
The fluorescence derived from EGFP-AoSec22 protein and *egfp-Aosec22* mRNA was observed in hyphal cells of the EGFP-AoSec22-expressing strain. **(A)** EGFP-AoSec22 was observed in live cells. **(B)** smFISH with *egfp* probe resulted in the observation of the fluorescence of EGFP-AoSec22 and *egfp-Aosec22* mRNAs. Nuclei were stained with DAPI. Bars, 10 μm. **(C)** Relative fluorescence intensity of *egfp-Aosec22* mRNA was determined at apical, middle, and basal cells. Bars show mean with standard error. Significant difference at *P* < 0.05 between a and b (Tukey–Kramer test, *n* = 10 cells).

**Figure 6:**
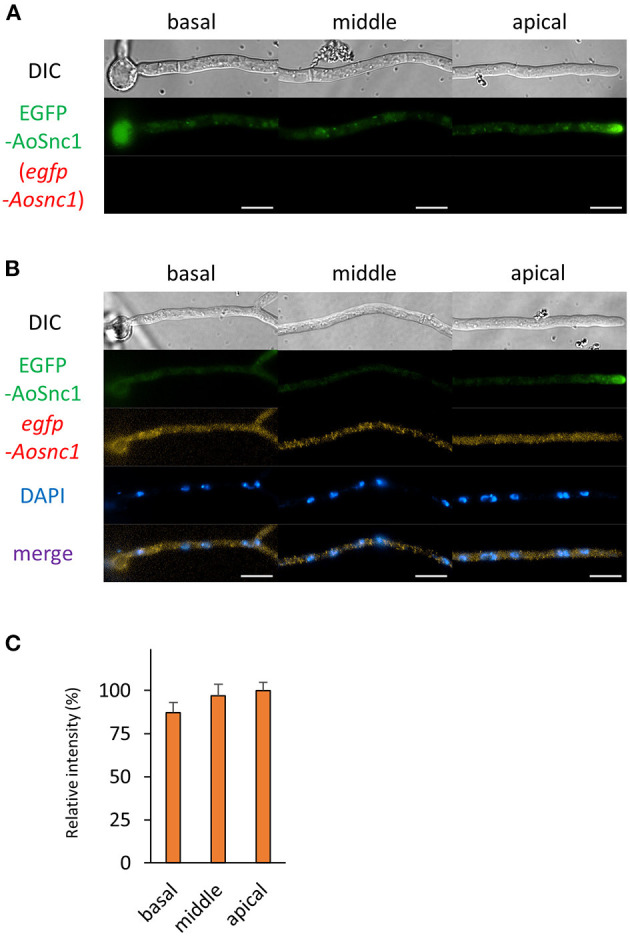
The fluorescence derived from EGFP-AoSnc1 protein and *egfp-Aosnc1* mRNA was observed in hyphal cells of the EGFP-AoSnc1-expressing strain. **(A)** EGFP-AoSnc1 was observed in live cells. **(B)** smFISH with *egfp* probe resulted in the observation of the fluorescence of EGFP-AoSnc1 and *egfp-Aosnc1* mRNAs. Nuclei were stained with DAPI. Bars, 10 μm. **(C)** Relative fluorescence intensity of *egfp-Aosnc1* mRNA was determined at apical, middle, and basal cells. Bars show mean with standard error (*n* = 10 cells).

Next, we analyzed the localization of mRNAs encoding proteins related to the secretory pathway. We generated strains expressing EGFP-fused AoSec22 or AoSnc1, SNARE proteins localized to ER or Spitzenkörper, respectively (Figures 5, 6 and Supplementary Figures 2C,D). smFISH using the *egfp* probe revealed that *egfp-Aosec22* mRNAs were localized through the cytoplasm of hyphal cells with a higher amount at the apical cell (Figure 5). In contrast, *egfp-Aosnc1* mRNAs were evenly distributed through the hyphal cells, although EGFP-AoSnc1 proteins were clustered at the tip region, forming Spitzenkörper (Figure 6). These results suggest that the localization and turnover rate of mRNA molecules is dependent on their nucleotide sequences.

### Localization of mRNA Encoding EGFP-Fused Vacuolar Membrane Protein

**Figure 7:**
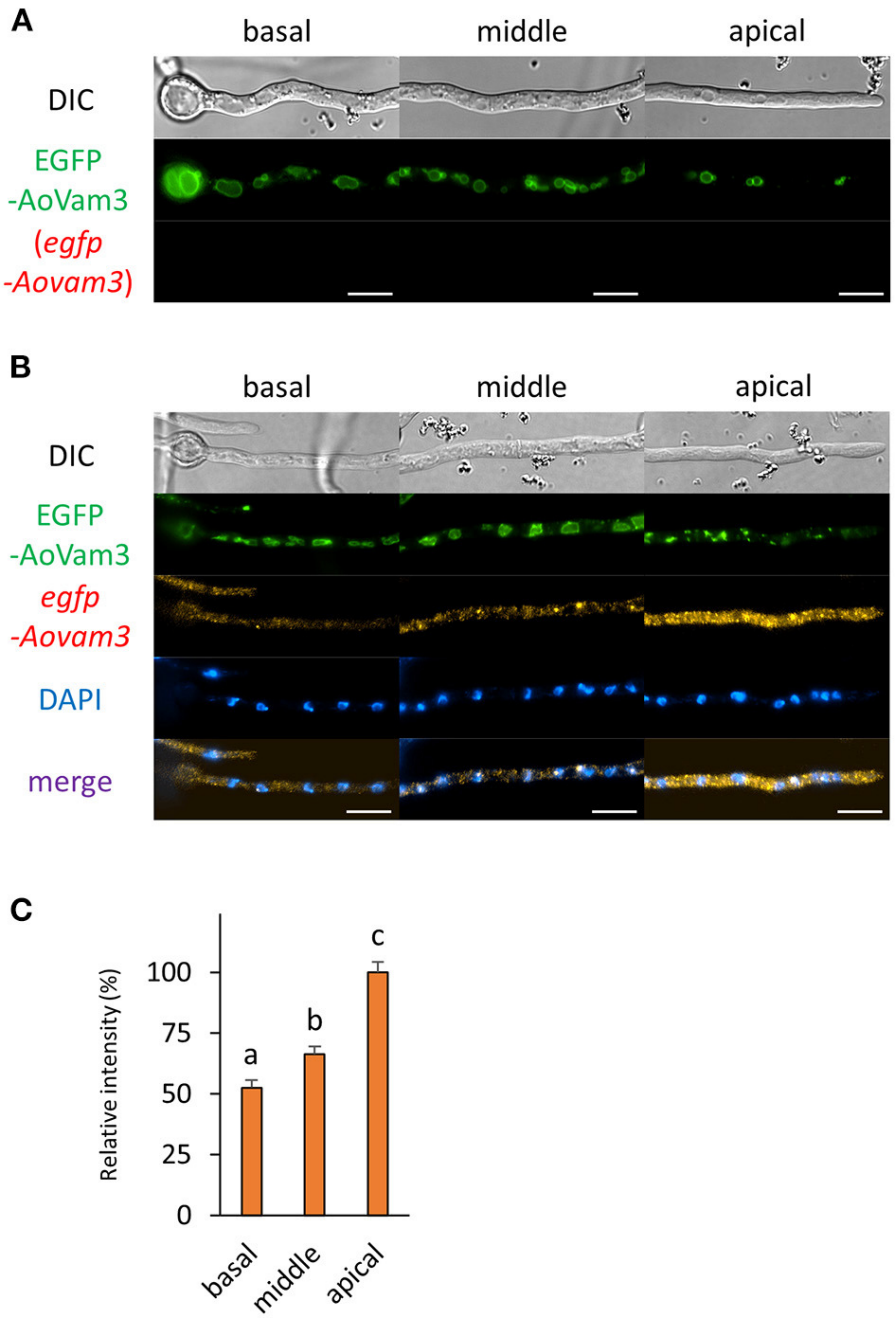
The fluorescence derived from EGFP-AoVam3 protein and *egfp-Aovam3* mRNA was observed in hyphal cells of the EGFP-AoVam3-expressing strain. **(A)** EGFP-AoVam3 was observed in live cells. **(B)** smFISH with *egfp* probe resulted in the observation of the fluorescence of EGFP-AoVam3 and *egfp-Aovam3* mRNAs. Nuclei were stained with DAPI. Bars, 10 μm. **(C)** Relative fluorescence intensity of *egfp-Aovam3* mRNA was determined at apical, middle, and basal cells. Bars show mean with standard error. Significant difference at *P* < 0.05 among each lowercase (Tukey–Kramer test, *n* = 10 cells).

Next, we wondered how mRNAs encoding proteins that mainly exist in the basal region were localized. We selected a vacuolar membrane SNARE protein AoVam3 and performed smFISH onto cells of the EGFP-AoVam3-expressiong strain (Shoji et al., 2006). As expected, the fluorescence of EGFP-AoVam3 was slightly observed in the apical cell where vacuoles are not well-developed (Figure 7 and Supplementary Figure 2E). In contrast to the localization pattern of EGFP-AoVam3, *egfp-Aovam3* mRNAs were mainly observed in the apical cell. These results suggest that *egfp-Aovam3* mRNAs are stably localized in the apical cell to be translated into proteins (Figure 7).

### Localization of mRNA Encoding EGFP-Fused Plasma Membrane Protein

**Figure 8:**
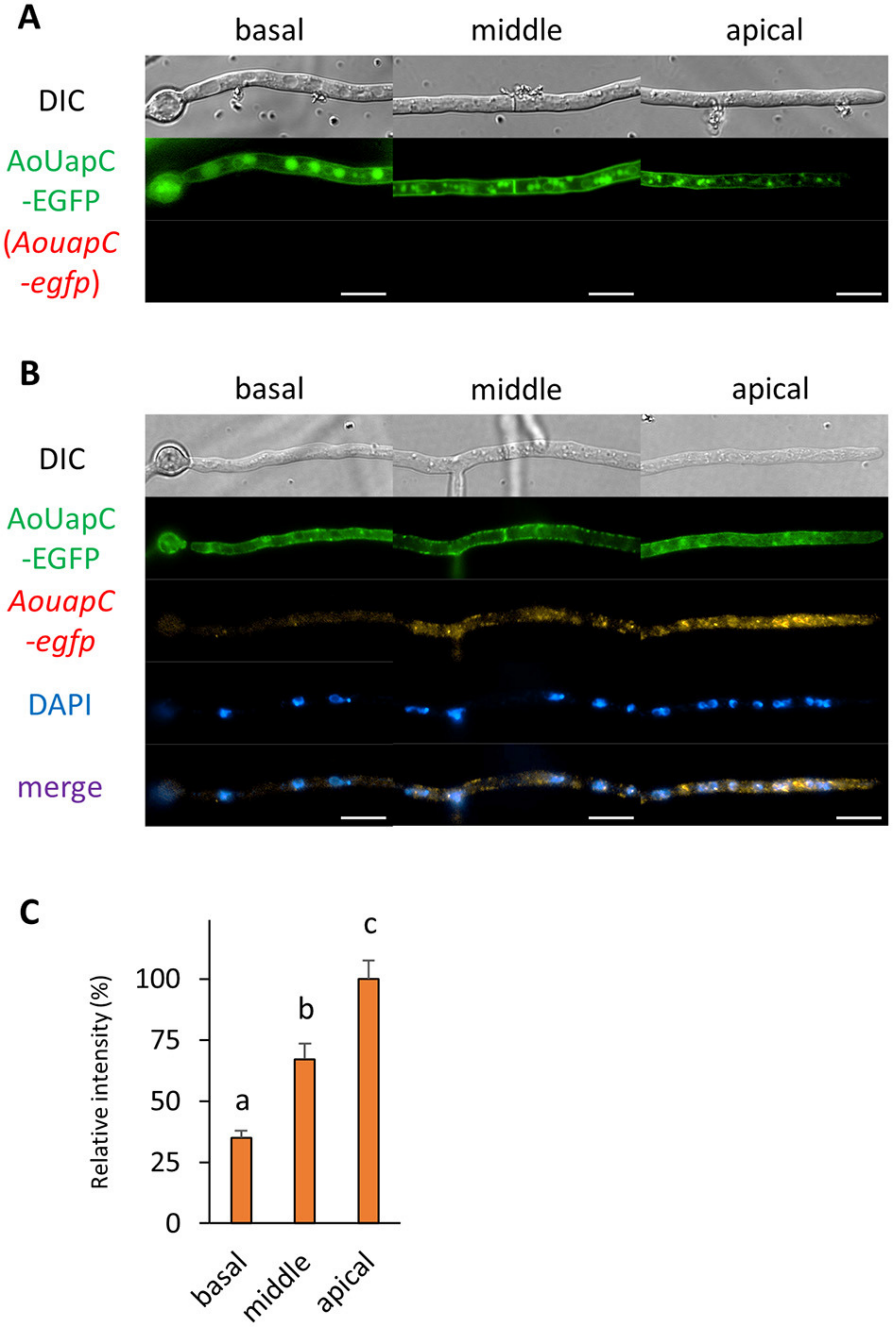
The fluorescence derived from AoUapC-EGFP protein and *AouapC-egfp* mRNA was observed in hyphal cells of the AoUapC-EGFP-expressing strain. **(A)** AoUapC-EGFP was observed in live cells. **(B)** smFISH with *egfp* probe resulted in the observation of the fluorescence of AoUapC-EGFP and *AouapC-egfp* mRNAs. Nuclei were stained with DAPI. Bars, 10 μm. **(C)** Relative fluorescence intensity of *AouapC-egfp* mRNA was determined at apical, middle, and basal cells. Bars show mean with standard error. Significant difference at *P* < 0.05 among each lowercase (Tukey–Kramer test, *n* = 10 cells).

Finally, we investigated the localization of mRNA encoding the plasma membrane purine transporter AoUapC (Higuchi et al., 2006). We performed smFISH onto the AoUapC-EGFP-expressing strain and found that the EGFP fluorescence in the vacuolar lumen was lost and that at the apical plasma membrane was more visible after smFISH procedures, likely because of permeabilization of vacuolar and plasma membrane, respectively (Figures 8A,B). However, the plasma membrane localization of AoUapC-EGFP was maintained even after smFISH procedures (Figure 8B and Supplementary Figure 2F). In cells expressing AoUapC-EGFP, we found that *AouapC-egfp* mRNAs were hardly seen in the basal cell, but highly observed in the apical cell (Figures 8B,C). These results suggest that *AouapC-egfp* mRNAs mainly exist in the apical region of the hyphal cell.

## Discussion

In this study, since we realized that the fluorescence and subcellular localization of EGFP-fused proteins were maintained even after the fixation of smFISH procedures in the hyphal cells of *A. oryzae*, we investigated the localization of mRNAs encoding EGFP-fused proteins by smFISH using an *egfp* probe. However, we found that smFISH procedures slightly affected certain EGFP localization; for example, the fluorescence of AoUapC-EGFP in the vacuolar lumen was lost after the fixation of smFISH. This might be caused by the steps of fixation and permeabilization on the membrane in smFISH. The mRNA localization patterns were distinct for cytoplasmic EGFP and EGFP-fused proteins, all of which were expressed under the same *amyB* promoter, suggesting that each mRNA localization was dependent on the transcript sequence of EGFP-fused proteins.

Since mRNA localization of actin cytoskeleton has been well-analyzed in a broad range of organisms (Buxbaum et al., 2015), we investigated mRNA localization of the EGFP-fused actin marker protein Lifeact by smFISH in the hyphae of *A. oryzae*. Previously, we revealed that mRNAs of actin-encoding *actA* are localized through the cell, especially at the hyphal tip region (Higuchi and Takegawa, 2020). Consistent with our previous smFISH result using an *actA* probe, we confirmed in this study by smFISH using an *egfp* probe that mRNAs encoding Lifeact-EGFP were also localized through the hyphal cell. Notably, the localization of both mRNAs and proteins of Lifeact-EGFP was observed at the hyphal tip, suggesting that mRNAs are transported to the hyphal tip and translated into proteins after being transcribed at the nuclei that are not localized around the hyphal tip region.

To obtain the information of mRNA localization of proteins related to the secretory pathway, we conducted smFISH with the hyphae of *A. oryzae* expressing EGFP-fused AoSec22 or AoSnc1, SNARE proteins localizing to ER or Spitzenkörper, respectively. mRNAs encoding EGFP-AoSec22 are preferentially localized to apical regions; in contrast, those encoding EGFP-AoSnc1 are almost evenly localized among hyphal regions. These results suggest that mRNAs encoding AoSec22 exist at the apical regions for *de novo* synthesis of AoSec22 proteins and that mRNAs encoding AoSnc1 might be translated into proteins evenly among hyphal regions. In mitotic cells of the yeast *Saccharomyces cerevisiae, SNC1* mRNAs are retained in mother cells, not exhibiting polarized localization, suggesting that Snc1 proteins are transported after being translated into proteins (Aronov et al., 2007). In contrast, in the hyphae of *A. oryzae*, it was reported that EGFP-AoSnc1 exhibits long-range bidirectional motility throughout the *A. oryzae* living cells, which is reminiscent of early endosome motility that is dependent on the microtubule, although its responsible motor proteins have not been identified yet in *A. oryzae* (Kuratsu et al., 2007; Higuchi, 2021b). This motility of AoSnc1 might reflect proteins being transported to be localized. Furthermore, AoSnc1 is thought to be recycled by endocytosis at the tip region (Higuchi et al., 2009a,b). Collectively, AoSnc1 proteins may be needed to be newly synthesized through the hyphal cells, which supports the ability of abundant protein secretion.

Although vacuoles, the membrane of which EGFP-AoVam3 localizes to, are highly formed at the basal region of the hyphae of *A. oryzae*, we found that *egfp-Aovam3* mRNAs were mainly localized at the apical cell. In plant cells, late endosomes and fragmented vacuoles are fused, resulting in large vacuoles (Cui et al., 2019). In *A. oryzae* cells, it may also be possible that small premature vacuoles in the apical cell would be fused to become large matured vacuoles in the basal region during hyphal development. Given that *de novo* AoVam3 proteins are produced in the apical cell, based on our observation of *egfp-Aovam3* mRNA localization, newly-synthesized AoVam3 proteins may function in membrane fusion of vacuole formation in the apical cell. To further reveal the molecular processes of vacuole formation, more correlative analyses on other mRNAs and proteins involved in these processes are needed.

We revealed that mRNAs encoding AoUapC-EGFP predominantly existed in the apical cell of the hyphae of *A. oryzae*. Similar purine transporter UapA in *Aspergillus nidulans* has been recently found to be secreted directly from ER to the plasma membrane, not via Golgi (Dimou et al., 2020). In addition, neosynthesized UapA is mainly secreted to the plasma membrane of the apical region during germling to hyphal transition, which is consistent with our observation of *AouapC-egfp* mRNA localization. Thus, most AoUapC proteins may be translated in the apical cell to get transported to the plasma membrane and stably localized throughout the hyphae of *A. oryzae*.

Our smFISH protocol using the *egfp* probe to investigate correlative localization of mRNA and protein is broadly applicable for, in principle, any EGFP-fused proteins. However, there is a limitation that the localization information obtained by smFISH is only from fixed cells, and thus, it is not possible to acquire when and where translation occurs for the target mRNA in living cells. To address this challenge, single-molecule imaging of translation has been successfully applied to living mammalian cells (Pichon et al., 2016; Wang et al., 2016; Wu et al., 2016; Yan et al., 2016; Mateju et al., 2020; Das et al., 2021). We are currently trying to introduce this live imaging system of mRNA translation into *A. oryzae* to reveal when, where, and how varieties of mRNA molecules are translated into proteins in the complex, multinuclear, and multicellular filamentous fungal cells.

## Data Availability Statement

The original contributions presented in the study are included in the article/Supplementary Material, further inquiries can be directed to the corresponding author.

## Author Contributions

YM performed the experiments. YM, YK, KT, and YH analyzed the data. YH wrote the manuscript and devised the project. All authors contributed to the article and approved the submitted version.

## Funding

This study was supported by JSPS KAKENHI grant number JP19H02874 (YH).

## Conflict of Interest

The authors declare that the research was conducted in the absence of any commercial or financial relationships that could be construed as a potential conflict of interest.

## Publisher's Note

All claims expressed in this article are solely those of the authors and do not necessarily represent those of their affiliated organizations, or those of the publisher, the editors and the reviewers. Any product that may be evaluated in this article, or claim that may be made by its manufacturer, is not guaranteed or endorsed by the publisher.
