# Supplementary material for: Correlative Localization Analysis Between mRNA and Enhanced Green Fluorescence Protein-Fused Protein by a Single-Molecule Fluorescence in situ Hybridization Using an egfp Probe in Aspergillus oryzae
Source: Front Fungal Biol. 2021 Oct 13;2:721398. doi: 10.3389/ffunb.2021.721398 (PMC10512357; doi:10.3389/ffunb.2021.721398)
Supplement: Supplementary file 1 [file Data_Sheet_1.PDF]

Supplementary Material

Correlative localization analysis between mRNA and EGFP-fused protein by single-molecule FISH using an *egfp* probe in *Aspergillus oryzae*

Yuki Morita, Yoshinori Katakura, Kaoru Takegawa, Yujiro Higuchi*

Department of Bioscience and Biotechnology, Faculty of Agriculture, Kyushu University, 744 Motooka, Fukuoka 819-0395, Japan

*Corresponding author. Tel/Fax: +81 92 802 4734, E-mail address: y.higuchi@agr.kyushu-u.ac.jp

Word count: Abstract, 178; Main, 3639.

Number of figures: Main, 8; Supplementary, 2.

Number of tables: Main, 1; Supplementary, 1.

Supplementary Table S1. smFISH probe for *egfp*.

Number	Sequence (5’-3’)	Position
Probe 1	ctcgcccttgctcaccat	1 bp
Probe 2	caccaccccggtgaacag	22 bp
Probe 3	tccagctcgaccaggatg	42 bp
Probe 4	ttgtggccgtttacgtcg	63 bp
Probe 5	ctcgccggacacgctgaa	82 bp
Probe 6	taggtggcatcgccctcg	102 bp
Probe 7	acttcagggtcagcttgc	122 bp
Probe 8	ttgccggtggtgcagatg	141 bp
Probe 9	gggccagggcacgggcag	160 bp
Probe 10	ttaacgtggtcacgaggg	179 bp
Probe 11	aagcactgcacgccgtag	198 bp
Probe 12	atgtggtcggggtagcgg	219 bp
Probe 13	gaagaagtcgtgctgctt	238 bp
Probe 14	cttcgggcatggcggact	257 bp
Probe 15	gtgcgctcctggacgtag	276 bp
Probe 16	gtcgtccttgaagaagat	295 bp
Probe 17	cgcgggtcttgtagttgc	314 bp
Probe 18	ccctcgaacttcacctcg	333 bp
Probe 19	gcggttcaccagggtgtc	352 bp
Probe 20	cgatgcccttcagctcga	371 bp
Probe 21	ttgccgtcctccttgaag	390 bp

Probe 22	cagcttgtgccccaggat	409 bp
Probe 23	gtggctgttgtagttgta	430 bp
Probe 24	cggccatgatatagacgt	449 bp
Probe 25	atgccgttcttctgcttg	468 bp
Probe 26	gatcttgaagttcacctt	487 bp
Probe 27	cgtcctcgatgttgtggc	506 bp
Probe 28	tcggcgagctgcacgctg	525 bp
Probe 29	ggtgttctgctggtagtg	544 bp
Probe 30	acggggccgtcgccgatg	564 bp
Probe 31	gtggttgtcgggcagcag	583 bp
Probe 32	cggactgggtgctcaggt	602 bp
Probe 33	ttggggtctttgctcagg	621 bp
Probe 34	catgtgatcgcgcttctc	640 bp
Probe 35	gtcacgaactccagcagg	660 bp
Probe 36	cgtgagtgatcccggcgg	680 bp
Probe 37	ttgtacagctcgtccatg	699 bp

In 717 b of *egfp* sequence, 37 regions of 18-22 nt were selected for the smFISH probe, each attached with CAL Fluor Red 610 fluorescence molecule.

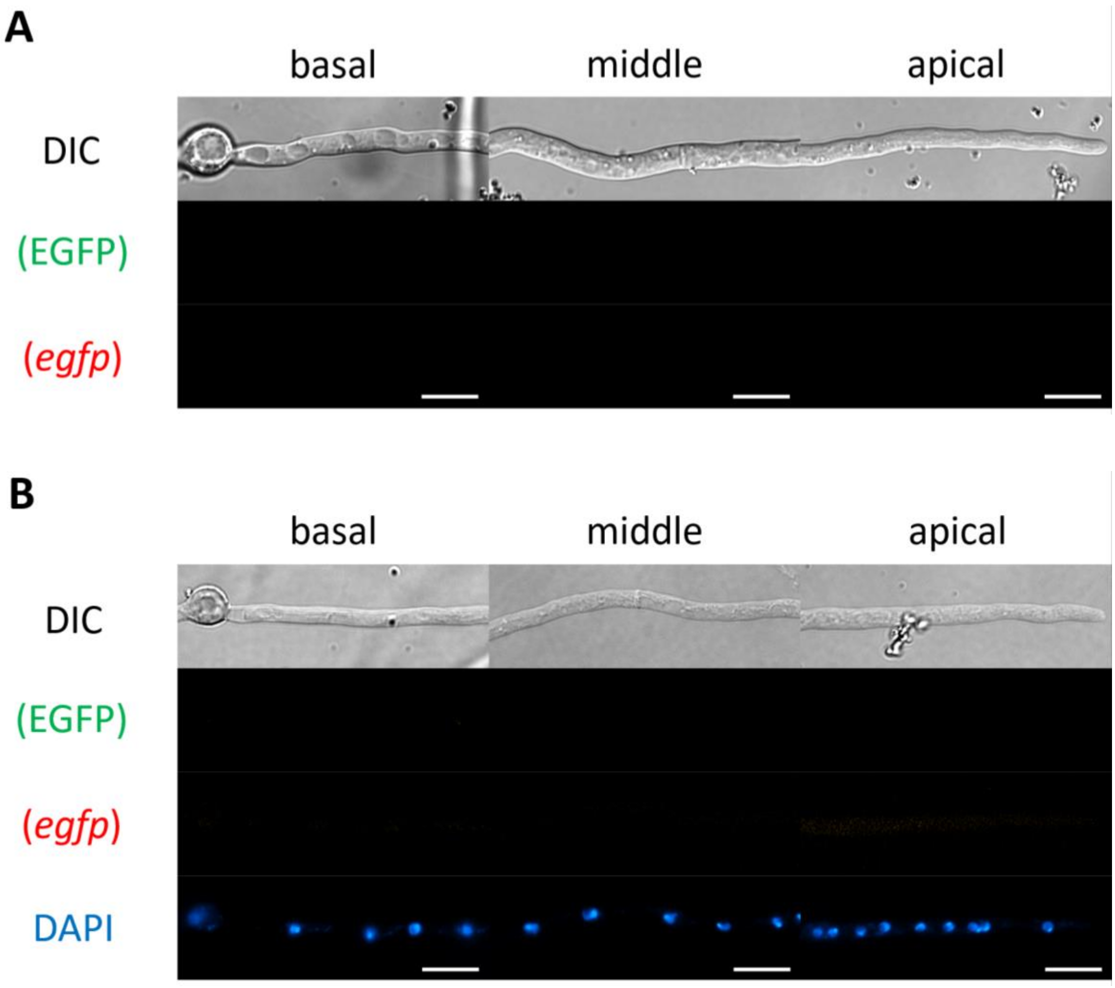

Supplementary Figure S1. Microscopic observation of hyphal cells of the wild-type RIB40 strain. No EGFP and red fluorescence appeared in live cells (**A**) and in cells after smFISH procedures without using the *egfp* probe (**B**). Nuclei were stained with DAPI. Bars, 10 μm.

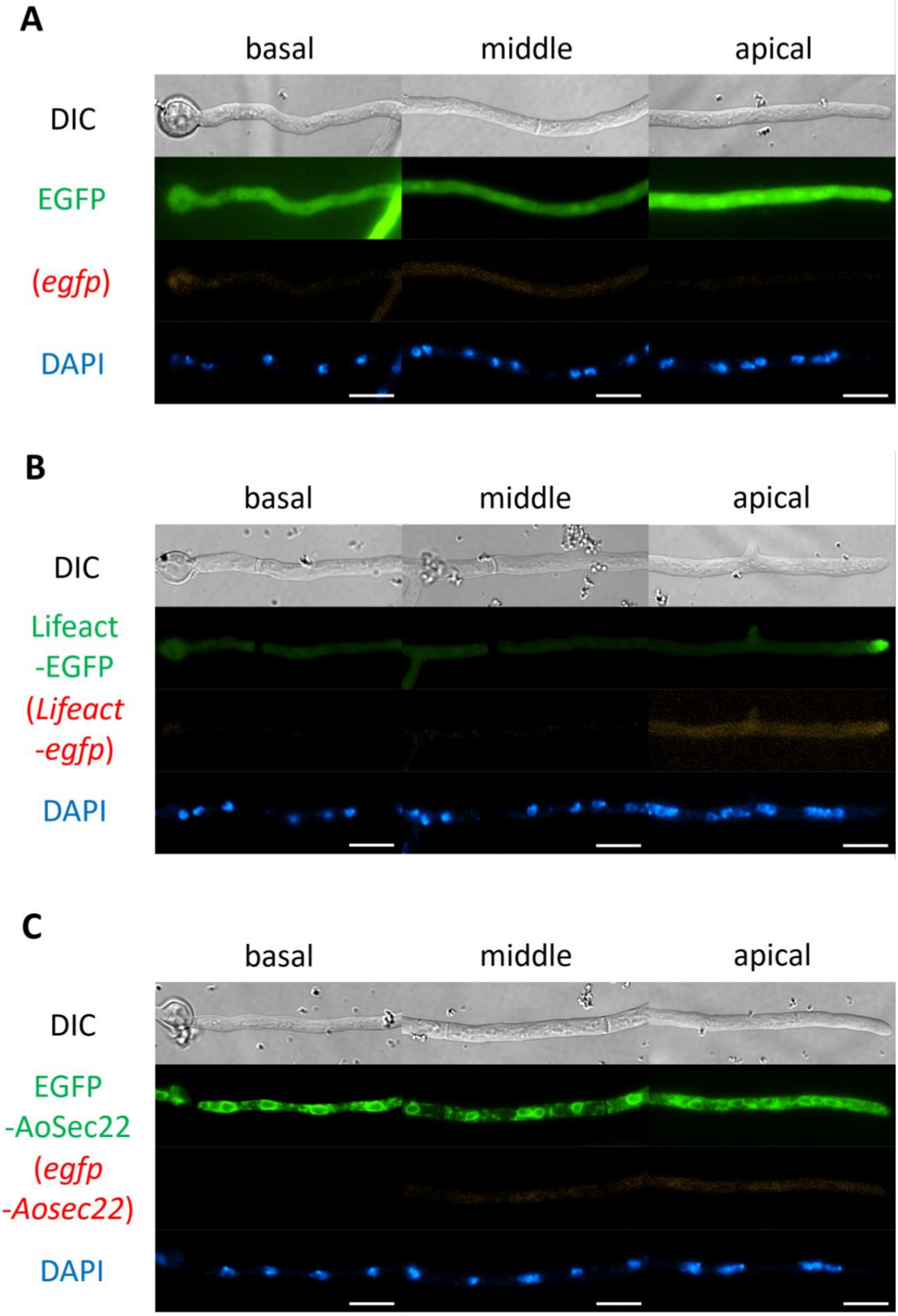
A
basal
middle
apical
DIC
EGFP
(egfp)
DAPI
B
basal
middle
apical
DIC
Lifeact
-EGFP
(Lifeact
-egfp)
DAPI
C
basal
middle
apical
DIC
EGFP
-AoSec22
(egfp
-Aosec22)
DAPI

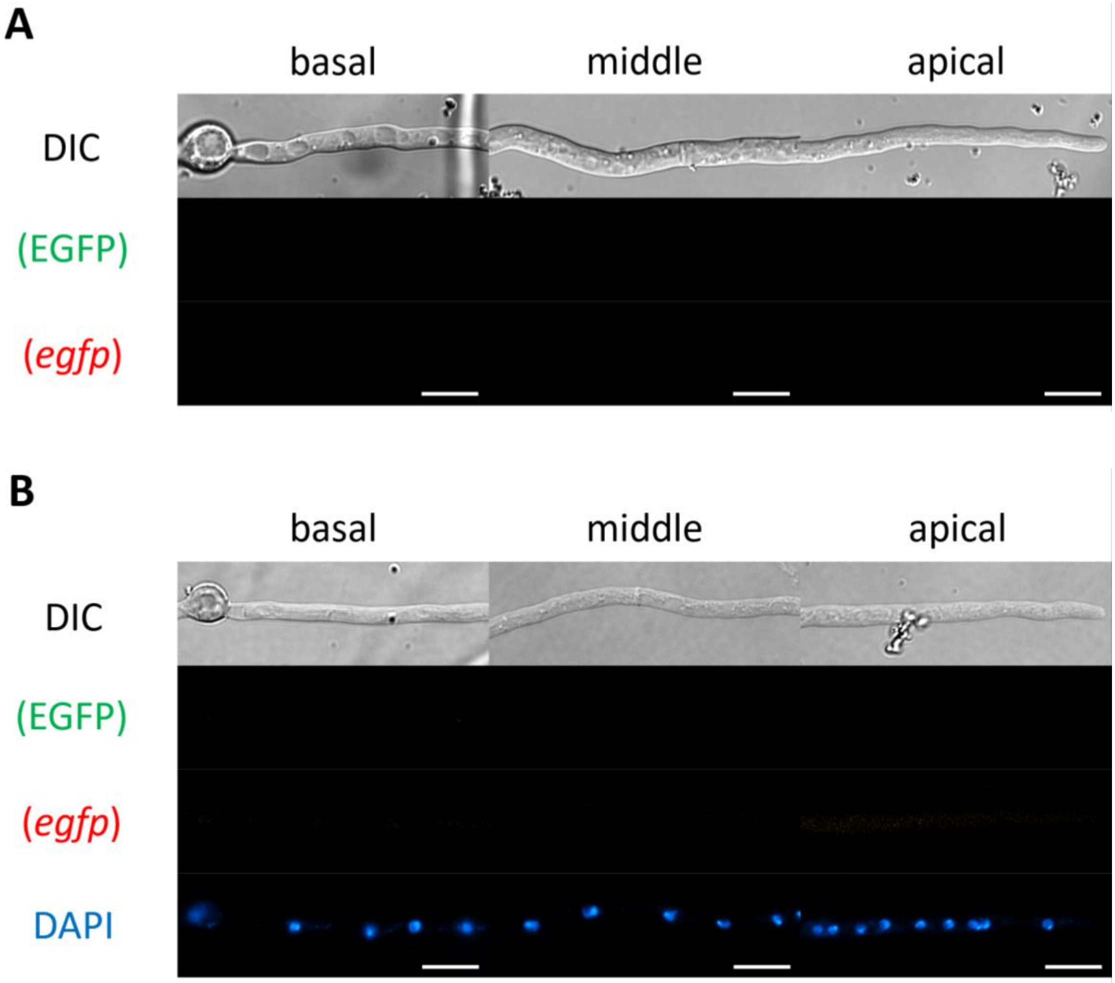

Supplementary Figure S1. Microscopic observation of hyphal cells of the wild-type RIB40 strain. No EGFP and red fluorescence appeared in live cells (**A**) and in cells after smFISH procedures without using the *egfp* probe (**B**). Nuclei were stained with DAPI. Bars, 10 μm.

Supplementary Figure S2. Microscopic observation of hyphal cells of strains expressing cytoplasmic EGFP or each EGFP-fused protein after smFISH procedures without using the *egfp* probe. EGFP fluorescence was observed in each cell expressing cytoplasmic EGFP (**A**), Lifeact-EGFP (**B**), EGFP-AoSec22 (**C**), EGFP-AoSnc1 (**D**), EGFP-AoVam3 (**E**) and AoUapC-EGFP (**F**). No red autofluorescence appeared. Nuclei were stained with DAPI. Bars, 10 μm.
